# Neuroplasticity in Early Onset Multiple Sclerosis

**Published:** 2014

**Authors:** Abdorreza Naser Moghadasi

**Affiliations:** Neuroscience institute, Sina Hospital, Tehran University of Medical Science, Tehran, Iran

## Introduction

Early onset multiple sclerosis (MS) refers to MS patients below 16 years of age that include 3–5% of MS cases (1) and have considerable differences with the adult type (2). In comparison to clinical features of adult MS, there is significant brain stem, cerebellum, and motor involvement in pediatric MS (3, 4). In addition, children have higher relapse rates than adults have. The relapsing-remitting type of MS (RRMS) is more frequent in children than primary progressive MS (PPMS) (2). Magnetic resonance imaging (MRI) findings of children are also different from an adult in that the MRIs will include several large plaques accompanied by gadolinium enhancing (2, 4). Meanwhile, despite acute features, disease symptoms recover well (2). In fact, children with MS demonstrate considerable recovery when compared with adults. However, despite its benign appearance, the overall prognosis is worse in children and the possibility of future disability is higher because the disease develops at early ages (3, 4). 

Neuroplasticity refers to a process through which the brain compensates for damages (5). It should be noted that neuroplasticity is a common phenomenon in MS patients and improves the condition. Additionally, it can be induced by physical and cognitive rehabilitation (6). Although new studies show that the relationship between age and neuroplastic response to brain injury is non-linear and complex (7); nevertheless, neuroplasticity emerges stronger and more effectively in children (5). Therefore, in spite of the acute and poly symptomatic emergence of MS in children, the significant recovery from symptoms occurs and neuroplasticity causes less disabilities that need to be rehabilitated (4). In spite of a good short-term prognosis for children, the longterm prognosis is not satisfactory and brings about more long-term disabilities (2, 3, 4). The reason may be due to an injury and subsequent neuroplasticity that the brain uses from its reserved capacity. Hence, brain capacity cannot be maintained as the patient grows older and experiences more complicated conditions, such as physical growth, greater practical use of muscles, and cognitive tests with more complex mental conditions. Actually, some kind of decompensation might have taken place. 

Perhaps, the unpleasant prognosis can be prevented by strengthening the physical and mental faculties of the patient. Since cognitive and physical rehabilitation can facilitate neuroplasticity (8), they should be considered as an important adjunctive therapy in early onset MS. 

In conclusion, researchers should respect the importance of neuroplasticity in early onset MS. The facilitation of this phenomenon can provide an injured brain with a better opportunity to recover.
